# Sinapine Modulates Glycogen and Lipid Synthesis via IRS1–PI3K–AKT–GSK3β–GS Pathway in Insulin‐Resistant Models

**DOI:** 10.1002/fsn3.71304

**Published:** 2026-01-04

**Authors:** Tiancheng Xing, Yiling Bai, Weijie Wu, Ziqi Zhao, Hanyu Kong, Qianyi Zhang, Shuoqi Li, Yan Liu, Xiaohui Guo, Zengli Wang

**Affiliations:** ^1^ College of Food Science and Nutritional Engineering China Agricultural University Beijing China; ^2^ Yingdong Intelligent Technology (Shandong) Co. Ltd. Jinan China

**Keywords:** glycogen synthesis, HepG2 cells, lipid synthesis, sinapine, T2DM

## Abstract

This study investigates the effects of sinapine on glycogen synthesis and lipid metabolism in insulin‐resistant HepG2 cell models and type 2 diabetes mellitus (T2DM) mice. Network pharmacology analysis integrated 288 potential sinapine targets and 920 insulin resistance‐related targets, yielding 72 overlapping genes. KEGG enrichment of these genes identified one significantly enriched insulin resistance pathway, with target mapping concentrated on the IRS1–PI3K–AKT–GSK3β–GS axis, suggesting a key role in promoting hepatic glycogen synthesis. Molecular docking identified these key targets on this signaling pathway, with sinapine showing strong binding affinity to its nuclear proteins (below −4.0 kcal/mol). In vitro, sinapine treatment improved glucose uptake and glycogen synthesis, while reducing lipogenesis, lipid accumulation, and reactive oxygen species (ROS) levels. RT‐qPCR and Western blot analyses confirmed that sinapine increases glycogen synthase activity. In T2DM mice, sinapine improved glucose and lipid metabolism, enhanced insulin sensitivity, and reduced blood glucose levels. Additionally, sinapine attenuated weight loss, improved liver index and histology, and regulated serum lipid profiles. Overall, this study reveals the molecular mechanism of sinapine in mitigating insulin resistance via modulation of the IRS1–PI3K–AKT–GSK3β–GS pathway, offering theoretical support for its potential application as a nutritional intervention to improve carbohydrate and lipid metabolism.

## Graphical abstract

This study combined network pharmacology, molecular docking, and in vitro (HepG2) and in vivo (T2DM mice) experiments to investigate sinapine's effect on insulin resistance. Sinapine was found to modulate the IRS1–PI3K–AKT–GSK3β–GS pathway. In vitro, sinapine improved glucose uptake, enhanced glycogen synthesis, and reduced lipogenesis and ROS. In vivo, sinapine improved glucose/lipid metabolism and insulin sensitivity, revealing its molecular mechanism for mitigating insulin resistance.

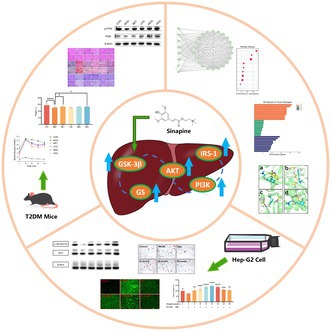

**Abbreviations**
AKT: protein kinase B
CCK‐8: Cell Counting Kit‐8
DMEM: Dulbecco's Modified Eagle Medium
ERK1/2: extracellular regulated protein kinases
G6Pase: glucose‐6‐phosphatase
GLUT2: glucose transporter type 2
GLUT4: glucose transporter type 4
GO: Gene Ontology
GS: glycogen synthase
GSK3: glycogen synthase kinase 3
GSK3β: glycogen synthase kinase 3β
HepG2: human hepatocellular carcinomas
INSR: insulin receptor
IR: insulin resistance
IRS: insulin receptor substrates
KEGG: Kyoto Encyclopedia of Genes and Genomes
mTOR: mammalian target of rapamycin
mTORC2: mammalian target of rapamycin complex 2
PDK1: 3‐phosphoinositide‐dependent protein kinase‐1
PEPCK: phosphoenolpyruvate carboxykinase
PI3K: phosphoinositide 3 kinase
PIP3: phosphatidylinositol 3,4,5‐trisphosphate
RT‐qPCR: real‐time quantitative reverse transcription polymerase chain reaction
Ser: serine
T2DM: type 2 diabetes mellitus
Thr: threonine
Tyr: tyrosine

## 1 Introduction

Patients with type 2 diabetes mellitus (T2DM) have metabolic abnormalities (Zheng et al. 2018) in glucose and lipid homeostasis (Pereira et al. 2021) that are primarily caused by insulin resistance (IR) (Li et al. 2022) and can lead to a number of serious complications (Bjornstad et al. 2023). At the heart of IR is a dysregulation of the insulin signaling pathway (Rohm et al. 2022) that impairs the sensitivity of target organs of insulin, particularly the liver (Saini 2010). Liver glycogen is a highly branched glucose polymer consisting of α‐particles formed by a series of interconnected β‐particles (Ryu et al. 2009). In T2DM, insulin resistance impairs the conversion of glucose into glycogen and prevents efficient storage. Meanwhile, changes occur in the key structure of glycogen, with α‐particles becoming more fragile and more susceptible to degradation into β‐particles (Jiang et al. 2016). The β‐particles are converted to glucose faster than the α‐particles due to their larger surface area, resulting in abnormal glucose release in T2DM patients (Wang et al. 2020). This disorder impairs the liver's ability to regulate and buffer blood glucose, which further impairs glucose homeostasis. Some evidence suggests that correcting these glycogen‐related abnormalities (López‐Soldado et al. 2021) and restoring hepatic glucose metabolism (Ros et al. 2011) may help alleviate insulin resistance and enhance insulin sensitivity in T2DM.

The pathological mechanisms of IR are complex. Previous studies have shown that insulin primarily exerts its effects in the liver through the phosphoinositide 3‐kinase (PI3K)/protein kinase B (Akt) signaling pathway (Rodgers et al. 2010). Additionally, activation of insulin receptor substrate 1 (IRS1) and its downstream PI3K/Akt pathway stimulates glucose uptake and reduces IR (Yang et al. 2018). Furthermore, the activation of AMP‐activated protein kinase (AMPK) can also suppress hepatic glucose production by regulating liver AMPK‐dependent mechanisms, such as inhibiting key enzymes involved in glucose production, including fructose‐1,6‐bisphosphatase (FBPase) and glucose‐6‐phosphatase (G6Pase) (Joshi et al. 2019). Under normal conditions, insulin activates IRS1 by binding to it, leading to the phosphorylation of IRS1, which in turn activates PI3K. PI3K subsequently phosphorylates Akt at the Ser473 site. Phosphorylated Akt (p‐Akt) regulates cellular metabolism by acting on downstream substrate molecules, reducing hepatic glucose production and glycogenolysis, while enhancing glycogen and fatty acid synthesis for storage and subsequent use (Taniguchi et al. 2006; Haeusler et al. 2018). However, in the presence of IR, the IRS1/PI3K/Akt signaling pathway fails to be activated. Damage to the IRS1/PI3K/Akt pathway leads to reduced hepatic glycogen synthesis, and consequently, Akt becomes a key therapeutic target for IR. Activating this signaling pathway can significantly improve the IR status of hepatocytes and alleviate hyperglycemia (Petersen and Shulman 2018; George et al. 2004).

Sinapine, a major secondary metabolite from cruciferous vegetables, has blood‐glucose‐lowering properties and can improve insulin sensitivity (Mouterde et al. 2020), although the precise mechanisms underlying these effects remain incompletely understood (Chu et al. 2024). In fact, while direct studies on sinapine are limited, sinapinic acid has been shown to restore both hepatic glycogen storage and enzyme activity in T2DM rats (Huang et al. 2022). In addition, there is evidence that sinapinic acid can inhibit apoptosis, DNA damage, and oxidative stress, upregulate antioxidant defense enzymes, and ameliorate structural and functional deterioration in the kidneys of diabetic rats (Altındağ and Özdek 2021).

Therefore, this study aims to systematically investigate how sinapine modulates hepatic glycogen synthesis and lipid metabolism in type 2 diabetes via the IRS1–PI3K–Akt–GSK3β–GS signaling pathway. We combined network pharmacology and molecular docking to predict key targets, followed by in vitro experiments in insulin‐resistant cells and in vivo validation in a T2DM mouse model (high‐fat diet and STZ‐induced), thus providing novel mechanistic insights for the nutritional application of sinapine in T2DM management.

## 2 Materials and Methods

### 2.1 Materials

All materials, reagents, instruments, and software tools used in this study are listed in detail in the Supporting Information. This includes cell lines, cell culture supplies, analytical reagents, assay kits, and software used for network pharmacology, molecular docking, and statistical analysis. Unless otherwise specified, all chemicals were of analytical grade and used without further purification (Tables S1 and S2). All animal experiments were conducted in accordance with ethical guidelines and approved by the Animal Welfare and Ethics Review Committee of China Agricultural University (Approval No. AW03504202‐5‐2).

### 2.2 Network Pharmacology Analysis and Molecular Docking Simulation

The canonical SMILES of sinapine was obtained from PubChem, and its structure file was imported into SwissADME to evaluate physicochemical properties, pharmacokinetics, and drug‐likeness. Target predictions were performed using BATMAN‐TCM, PharmMapper, and SwissTargetPrediction. Insulin resistance‐related genes were retrieved from TCMSP, HERB, OMIM, DrugBank, GeneCards, TTD, and DisGeNET, and overlapping targets with sinapine were identified. STRING was used to construct a protein–protein interaction (PPI) network for *Homo sapiens* , and Cytoscape v3.10.2 was employed to analyze network topology (Table S3).

Functional enrichment analysis (GO and KEGG) was conducted using the clusterProfiler and pathview packages in R (v4.5.1). The organism was set to *H. sapiens* . Enrichment significance was evaluated using a hypergeometric test, and *p*‐values were adjusted using the Benjamini–Hochberg method to control the false discovery rate (FDR). GO terms with adjusted *p* < 0.05 were considered statistically significant, and a target‐pathway network was visualized with Cytoscape.

Key targets involved in hepatic glycogen synthesis were selected for molecular docking. Protein structures were retrieved from RCSB PDB, preprocessed (removal of water/ions, hydrogen addition, charge assignment), and docked with sinapine using CB‐DOCK2, which performed automated template‐based or blind docking. AutoDock Vina ranked binding sites based on Vina scores (kcal/mol), assessing sinapine's affinity for target proteins.

### 2.3 Cell Culture

When cells reached over 80% confluence in the culture flask, cells were subcultured. In a biosafety cabinet, medium was removed and cells were washed twice with 1 mL phosphate‐buffered saline (PBS). Then, 1 mL of trypsin–EDTA was added for digestion. When the cells appeared as bright, round spheres under the microscope with cytoplasmic retraction, 1 mL of DMEM was added to terminate digestion, followed by gentle pipetting to ensure single‐cell suspension. Cells were transferred to a centrifuge tube and centrifuged at 4°C, 1000 rpm for 5 min. After discarding the supernatant, 1 mL of complete medium was added to resuspend the cells. The suspension was evenly distributed into new flasks and supplemented with DMEM to a final volume of 5 mL. The flasks were sealed, labeled, disinfected, and returned to the incubator (37°C, 5% CO_2_) for further culture.

### 2.4 Insulin Resistance Model (IR)

Log‐phase HepG2 cells were used for IR modeling. Insulin (INS) and palmitic acid (PA) co‐treatment was used to induce IR. Cells were seeded at a density of 5 × 10^3^ cells per well in a 96‐well culture plate, with one control and five treatment groups (*n* = 8 wells/group). After 24 h of culture, the intervention substances were added. The empty control group received only DMEM, while the model groups were supplemented with INS and PA according to the experimental design and continued the culture for another 24 h. All intervention and modeling substances were added to the culture medium in advance to minimize the operating time and reduce possible effects on the cells (Yudhani et al. 2023).

### 2.5 Cell Viability

100 L of DMEM containing 10% CCK‐8 was added to each well of the 96‐well plate. The plate was incubated in the incubator for 1 h, ensuring that the incubation time was controlled to achieve absorbance in the range of 0.5–0.8. The absorbance at 450 nm was measured using a microplate reader (Fang et al. 2024).

### 2.6 Glucose Uptake/Consumption

Log‐phase HepG2 cells were seeded at 5 × 10^3^ cells/well in 96‐well plates, with one control group and five sinapine‐treated model groups (*n* = 8 wells/group). After 24 h of culture, insulin resistance was induced (INS + PA). Following successful modeling, cells were treated with high‐glucose DMEM containing different concentrations of sinapine, while the control and model groups received DMEM only. After 24 h of intervention, the supernatant was collected, and glucose concentration (mg/dL) was measured using a glucose assay kit. Glucose consumption was calculated as the difference between the glucose concentration in the medium incubated without cells and the experimental supernatant. Glucose uptake was normalized to cell viability (Nie et al. 2017).

### 2.7 Glycogen Content

Log‐phase HepG2 cells were seeded into six‐well plates and divided into a blank control group, a model group, and four sinapine treatment groups. After 24 h of culture, IR was induced. Following successful modeling, cells were treated with high‐glucose DMEM containing different concentrations of sinapine for another 24 h, while the control and model groups received DMEM only. Cells were collected and centrifuged to remove the supernatant. A total of 0.75 mL extraction buffer was added to the cell pellet, and lysis was performed via ultrasonication (200 W, 3 s on/10 s off, 30 cycles). The lysate was transferred to a 10 mL tube and boiled for 20 min with the lid closed, mixing three times every 5 min. After cooling to room temperature, the volume was adjusted to 5 mL with distilled water, mixed, and centrifuged at 8000*g* for 10 min at room temperature (≈25°C). The glycogen content in the supernatant was determined using a commercial assay kit according to the manufacturer's instructions (Zhang et al. 2013).

### 2.8 Cellular Lipid Distribution and Content

Oil Red O stock solution was prepared by dissolving 0.5 g of Oil Red O powder in 100 mL isopropanol. For staining, the stock solution was mixed with double‐distilled water at a 3:2 (v/v) with ddH_2_O and filtered through a 0.45 μm membrane to obtain the working solution. Log‐phase cells were seeded in six‐well plates, and an IR model was established. After 24 h of treatment, cells were washed twice with PBS and fixed in 4% paraformaldehyde for 15 min. Following fixation, cells were stained with 1 mL Oil Red O working solution at room temperature for 15 min and washed with PBS until no residual dye was visible. Cellular morphology and lipid droplet accumulation were observed and imaged under a microscope. For quantification of intracellular lipid content, stained cells were incubated with isopropanol to extract the dye. The extracted solution was transferred to a 96‐well plate, and absorbance at 490 nm was measured using a microplate reader (Li et al. 2021).

### 2.9 Reactive Oxygen Species (ROS)

HepG2 cells in log phase were seeded into six‐well plates and cultured for 24 h. After successful establishment of the insulin resistance model, cells were treated with high‐glucose DMEM containing different concentrations of sinapine. The control, model, and positive control groups received DMEM only. After 24 h of treatment at 37°C with 5% CO_2_, the medium was discarded and the cells were washed twice with PBS. A working solution of DCFH‐DA (1:1000 dilution in serum‐free medium) was added (1 mL/well), and cells were incubated at 37°C for 30 min. The probe solution was removed, and cells were washed 2–3 times with PBS to eliminate excess dye. PBS was added to maintain moisture, and fluorescence was observed using a fluorescence microscope (excitation: 488 nm; emission: 525 nm) (Qiao et al. 2013).

### 2.10 Gluconeogenic Enzyme Activity

HepG2 cells in log phase were seeded into six‐well plates and cultured for 24 h. Following insulin resistance induction, cells were treated with DMEM containing various concentrations of sinapine, while control, model, and positive control groups received DMEM only. After 24 h of incubation at 37°C with 5% CO_2_, the activities of phosphoenolpyruvate carboxykinase (PEPCK) and glucose‐6‐phosphatase (G6Pase) were measured using commercial assay kits, according to the manufacturers' protocols (Shen et al. 2012).

### 2.11 Western Blot In Vitro Experiments

After treatment (Section 2.3), cells were washed twice with pre‐chilled PBS and lysed using RIPA buffer containing protease inhibitors (PI:RIPA = 1:100). The lysate was sonicated (20 W) and centrifuged (15,000 rpm, 4°C, 30 min) to obtain the supernatant. Protein concentration was quantified using a BCA kit and normalized with loading buffer. Samples were denatured at 100°C for 5 min and stored at −80°C. Equal amounts of protein were separated by 15% SDS‐PAGE and transferred to PVDF membranes. Membranes were blocked with 5% nonfat milk for 1 h, incubated with primary antibodies at 4°C overnight, then with secondary antibodies at 37°C for 1 h. Protein bands were visualized using ECL reagents and quantified using β‐actin as an internal control (Mao et al. 2024).

### 2.12 RNA Extraction and RT‐qPCR

Total RNA was extracted using TRIzol reagent. RNA purity and concentration were assessed with a NanoDrop 2000 spectrophotometer.

cDNA was synthesized using the PrimeScript RT Reagent Kit. RT‐qPCR was conducted using TBGreen Fast qPCR Mix with cDNA as the template. Each sample was analyzed in triplicate. Relative gene expression was calculated using the 2^−ΔΔCt^ method.

### 2.13 Animal Model Establishment and Intervention Treatment

Fifty male C57BL/6J mice (4 weeks old, 20–25 g) were acclimated for 1 week and randomly divided into a standard diet group (*n* = 10) and a high‐fat diet (HFD) group (*n* = 40). After 4 weeks of HFD feeding, mice in the HFD group were intraperitoneally injected with 60 mg/kg streptozotocin (STZ) for three consecutive days. One week after modeling, mice with sustained fasting blood glucose (FBG) levels ≥ 11.1 mmol/L were considered to have developed T2DM. These mice then underwent a 6‐week intervention: the control group (CON) received a standard diet and saline; the model group (MOD) continued on the HFD and saline; the positive control group (MET) received 200 mg/kg metformin; and the sinapine‐treated groups (LDG, MDG, HDG) received 50, 100, and 200 mg/kg sinapine, respectively. Gavage was performed daily at 10:00 a.m., and body weight and 8‐h FBG were recorded weekly.

### 2.14 Oral Glucose Tolerance Test (OGTT) and Insulin Tolerance Test (ITT)

After a 6‐h fast, mice underwent OGTT; on a separate day, after a 6‐h fast, mice underwent ITT. In the OGTT, mice were gavaged with 2.0 g/kg glucose solution in saline; in the ITT, they were intraperitoneally injected with 0.5 U/kg insulin aspart. Blood glucose levels were measured at 0, 30, 60, 90, and 120 min post‐administration. Glucose response curves were plotted, and the area under the curve (AUC) was calculated.

### 2.15 Serum Collection and Liver Index Measurement

Blood samples were collected via retro‐orbital blood collection and allowed to clot at room temperature for 30 min. Samples were centrifuged at 4°C, 3000 rpm for 15 min, and serum was collected. After dissection, the liver was excised, rinsed with saline, blotted dry, and weighed.

### 2.16 Biochemical and Histological Analysis

Serum levels of triglycerides (TG), total cholesterol (TC), high‐density lipoprotein cholesterol (HDL‐C), low‐density lipoprotein cholesterol (LDL‐C), and glycosylated serum protein (GSP) were measured using commercial kits, according to the manufacturers' instructions. Liver tissues from the same anatomical region were stained with Oil Red O to evaluate lipid accumulation. For hepatic glycogen measurement, 20 mg of liver tissue was homogenized in nine volumes of PBS to prepare a 10% homogenate, followed by detection based on kit instructions.

### 2.17 Western Blot Analysis of Liver Tissue

Liver tissue samples were lysed in buffer (1 mL per 200 mg tissue), homogenized on ice in RIPA buffer, and centrifuged at 12,000 rpm for 10 min at 4°C. The supernatant was collected, protein concentrations were measured, and samples were adjusted to a loading amount of 51–100 μg. Protein samples were mixed with 5× loading buffer at a 1:4 ratio, boiled for 10 min, and stored at −80°C. Proteins were separated by SDS‐PAGE and transferred onto PVDF membranes. Membranes were blocked with 5% skim milk for 1 h at room temperature, incubated overnight with primary antibodies at 4°C, and then with secondary antibodies at 37°C for 1 h. Chemiluminescent signals were detected using an ECL kit and imaged.

### 2.18 Statistical Analysis

Western blot band intensity was quantified using ImageJ to obtain integrated optical density (IOD) values, which were normalized to the corresponding loading controls. All results were expressed as the mean ± SEM based on six biological replicates, each measured in triplicate. GraphPad Prism 9.0.0 and IBM SPSS Statistics 26 were used for data analysis. Comparisons between two groups were conducted using unpaired *t*‐tests, while one‐way ANOVA followed by Tukey's post hoc test was used for multiple‐group comparisons.

## 3 Results

### 3.1 Identification of Key Targets and Pathways of Sinapine in the Insulin Resistance Signaling Network

**FIGURE 1:**
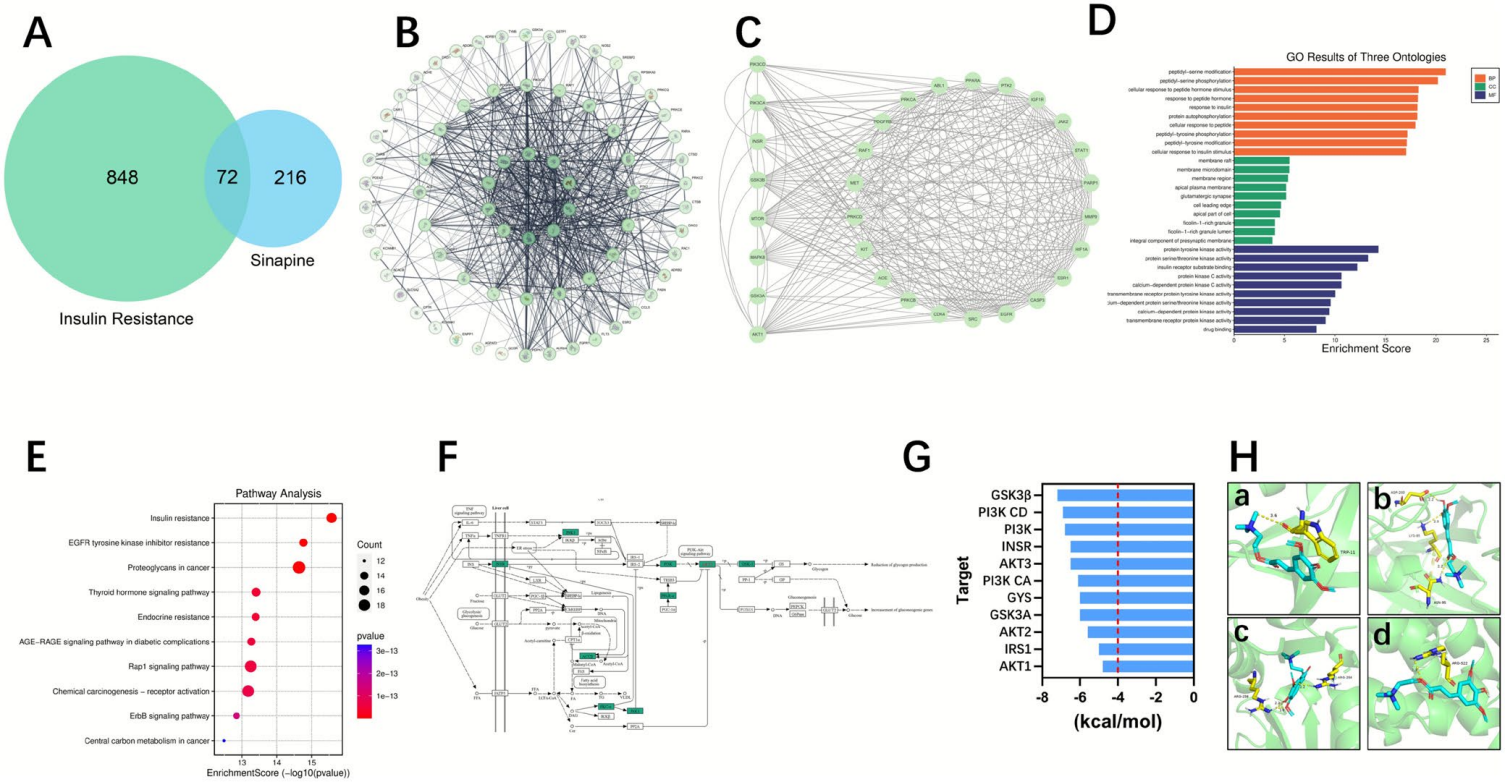
Network pharmacology and molecular docking analysis of the potential mechanisms of sinapine in insulin resistance. (A) Venn diagram showing the overlapping targets of sinapine and insulin resistance; (B) Protein–protein interaction (PPI) network constructed from overlapping targets; (C) Optimized PPI network visualizing the top 30 targets ranked by degree value, with low‐connectivity nodes removed; (D) Gene Ontology (GO) enrichment analysis of the potential targets; (E) KEGG pathway enrichment analysis of the common targets; (F) Enrichment map of key targets in the insulin resistance signaling pathway; (G) Binding energies from molecular docking between sinapine and key proteins involved in glycogen synthesis. All binding energies were below −4 kcal/mol, indicating potential binding affinity; (H) Predicted binding conformations of sinapine with core proteins in the insulin signaling pathway: a, AKT1; b, GSK3β; c, IRS1; d, PI3K.

A total of 288 potential sinapine targets were identified by integrating 63 targets from BATMAN‐TCM, 129 from PharmMapper, and 110 from SwissTargetPrediction. Meanwhile, 920 insulin resistance‐related targets were collected from TCMSP, HERB, OMIM, DrugBank, GeneCards, TTD, and DisGeNET. Venn analysis revealed 72 overlapping genes (25% of sinapine targets), as shown in Figure 1A (Tables S4 and S5).

These overlapping genes formed a protein–protein interaction (PPI) network with 619 edges (average 17.2 edges per node, clustering coefficient 0.637, *p <* 10^−16^) (Figure 1B). After filtering low‐connectivity nodes, the core PPI network (Figure 1C) included 30 nodes and 447 edges. AKT1, PI3K, MTOR, GSK3β, MAPK8, and INSR ranked among the most central nodes within this core network.

GO enrichment identified key biological processes, cellular components, and molecular functions (Figure 1D). In the GO‐CC enrichment results, terms such as membrane raft, membrane microdomain, apical plasma membrane, cell leading edge, glutamatergic synapse, and integral component of presynaptic membrane may at first seem unrelated to insulin resistance. However, many of these annotations stem from prior studies in neural or epithelial tissues, and in insulin‐responsive cells they likely reflect more general roles in membrane compartmentalization, receptor localization, vesicle trafficking, or calcium‐dependent signaling. Such functions can affect receptor internalization, spatial proximity to downstream kinases, and membrane‐vesicle exchange of signaling molecules, thereby influencing insulin signaling. Enrichment in ficolin‐1‐rich granule further suggests links to immune/acute‐phase responses, consistent with metabolic inflammation in insulin resistance. Crucially, the BP and MF enrichments (e.g., peptidyl phosphorylation, receptor tyrosine kinase activity) directly align with our experimental findings on the IRS1–PI3K–AKT–GSK3β–GS axis. Taken together, these GO results support the hypothesis that sinapine improves hepatic insulin signaling by modulating membrane localization and kinase activity, ultimately ameliorating glucose and lipid metabolic dysregulation (Table S6). KEGG pathway analysis (Figure 1E) highlighted significant enrichment in the insulin resistance pathway. In this pathway, sinapine targets were enriched in the IRS1–PI3K–AKT–GSK3β–GS axis, suggesting a potential role in enhancing glycogen synthesis (Figure 1F). Molecular docking confirmed stable binding between sinapine and four core targets, all with binding energies < −4 kcal/mol (Figure 1G). Hydrogen bonding was observed between sinapine and key residues: TRP‐11 (AKT), ASP‐200/LYS‐85/ASN‐95 (GSK3β), ARG‐258 (IRS1), and ARG‐522 (PI3K) (Figure 1H), indicating potential regulatory effects on phosphorylation and activity of these proteins.

### 3.2 Effects of Sinapine on Cell Viability and Glucose Metabolism in Insulin‐Resistant HepG2 Cells

**FIGURE 2:**
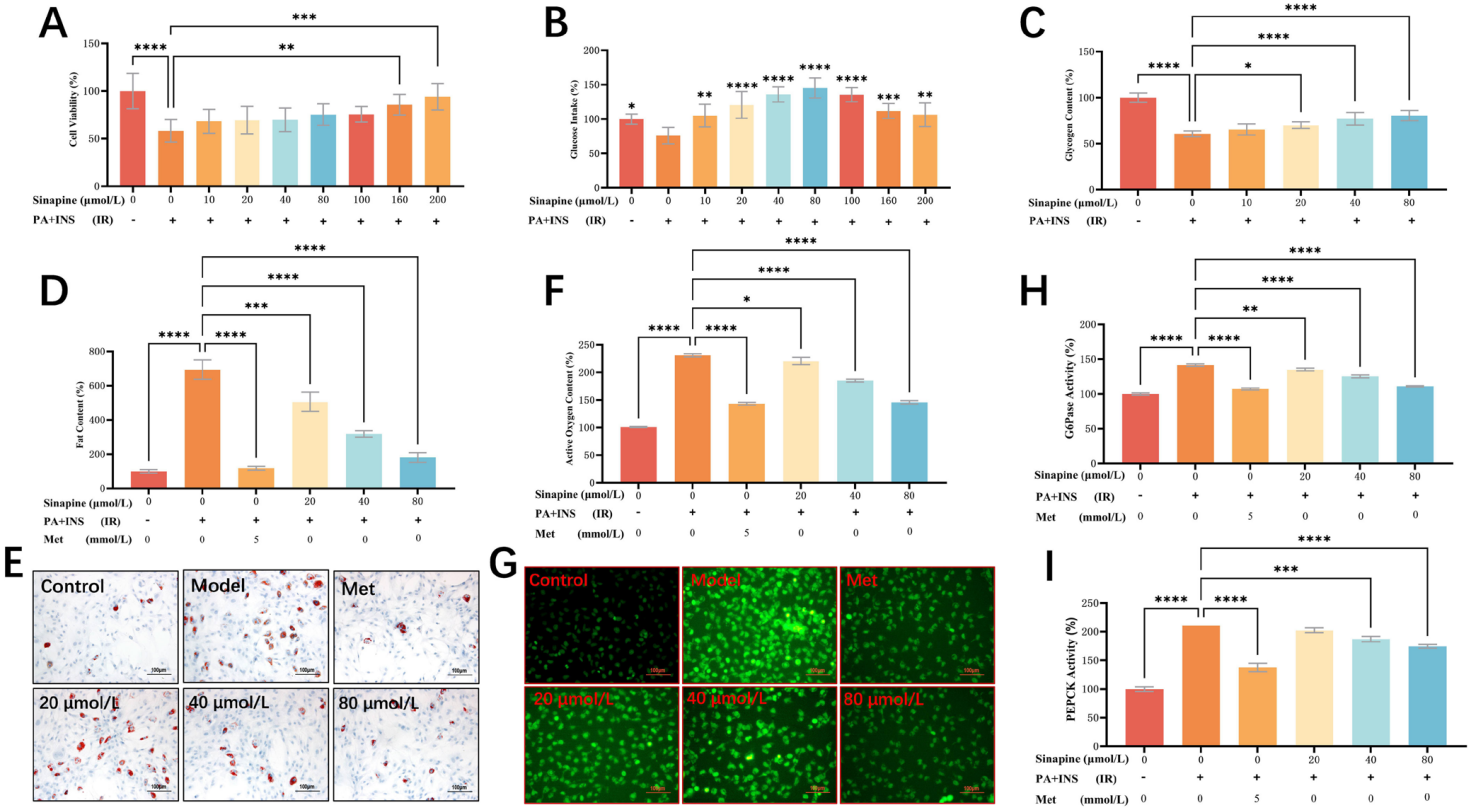
Effects of sinapine on glucose metabolism, lipid accumulation, oxidative stress, and gluconeogenesis in insulin‐resistant HepG2 cells. (A) Cell viability after treatment with different concentrations of sinapine; (B) Glucose uptake under sinapine intervention; (C) Intracellular glycogen content after sinapine treatment; (D) Quantification of lipid accumulation based on Oil Red O staining; (E) Representative Oil Red O staining images showing intracellular lipid accumulation; (F) Quantification of intracellular ROS levels; (G) Distribution of intracellular reactive oxygen species detected by DCFH‐DA staining; (H, I) Enzymatic activities of gluconeogenesis‐related enzymes G6Pase and PEPCK under different concentrations of sinapine.(*p < 0.05, **p < 0.01, ***p < 0.001, ****p < 0.0001).

Sinapine treatment at 10–200 μmol/L for 24 h improved the viability of insulin‐resistant HepG2 cells, with 160 and 200 μmol/L groups showing significant increases (85.75% ± 4.43% and 94.14% ± 5.68%) compared to the model control (58.25% ± 4.82%, *p* < 0.05; Figure 2A). Glucose uptake, sinapine at 10–200 μmol/L significantly enhanced uptake capacity (*p* < 0.005), peaking at 80 μmol/L (145.30% ± 5.93%; Figure 2B). Significant increases in glycogen synthesis were observed starting at 20 μmol/L (*p* < 0.05), while 40 and 80 μmol/L showed highly significant increases (*p* < 0.0001; Figure 2C).

### 3.3 Effects of Sinapine on Lipid Metabolism in Insulin‐Resistant HepG2 Cells

Oil Red O staining revealed that sinapine at 20–80 μmol/L for 24 h reduced intracellular lipid droplet accumulation in insulin‐resistant HepG2 cells, comparable to the effect of 5 mmol/L metformin. The 20 μmol/L group (506.30% ± 32.48%) showed a significant reduction versus the model control (693.80% ± 32.48%, *p* < 0.001), while the 40 μmol/L (318.80% ± 10.83%) and 80 μmol/L (181.30% ± 16.54%) groups had even greater reductions (*p* < 0.0001; Figure 2D), indicating a significant reduction in intracellular lipid content (Figure 2E).

### 3.4 Effects of Sinapine on Oxidative Stress and Gluconeogenesis Enzyme Activity

Sinapine treatment significantly reduced ROS levels in all groups compared with the model control. The 20 μmol/L group showed a level of 220.70% ± 3.71% (vs. 231.00% ± 1.73%, *p* < 0.05), while the 40 and 80 μmol/L groups had ROS levels of 185.3% ± 1.45% and 146% ± 1.74%, respectively (*p* < 0.0001; Figure 2F,G). Gluconeogenesis‐related enzyme activity decreased after sinapine intervention. G6Pase activity in the 20, 40, and 80 μmol/L groups was significantly reduced (135.40%, 125.40%, 111.20%, *p* < 0.01; Figure 2H) and PEPCK activity in the 40 and 80 μmol/L groups was markedly decreased (187.30%, 174.60%, vs. 210.00%, *p* < 0.001). The 20 μmol/L group also showed lower activity (202.60%; Figure 2I).

### 3.5 mRNA Expression of Insulin Resistance‐Related Genes in Sinapine‐Treated HepG2 Cells

**FIGURE 3:**
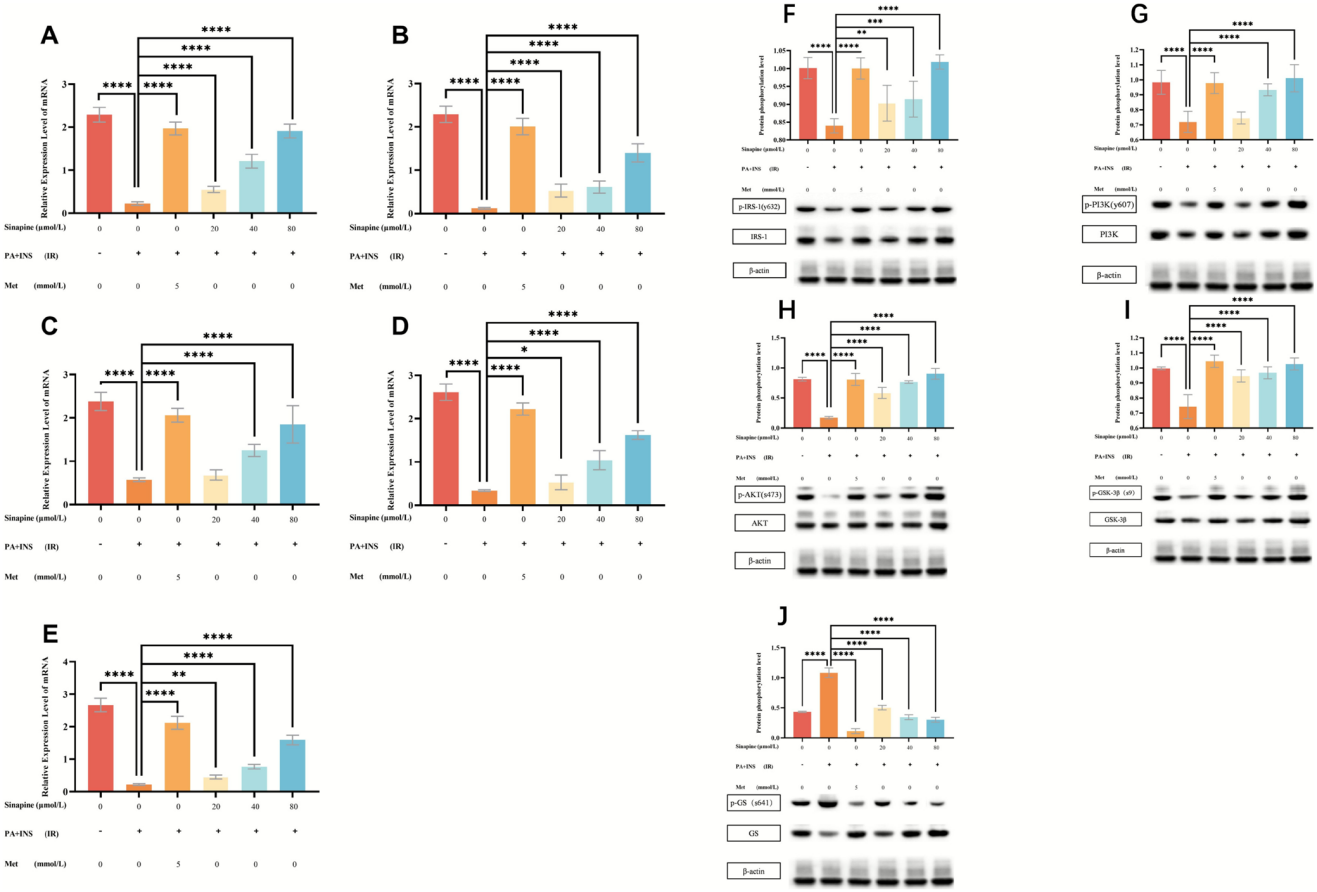
Effects of sinapine on gene expression and protein phosphorylation in the insulin resistance pathway in IR‐HepG2 cells. (A–E) Relative mRNA expression levels of IRS1, PI3K, AKT, GSK3β, and GS analyzed by RT‐qPCR. (F–J) Phosphorylation levels of IRS1 (Tyr632), PI3K‐p85 (Tyr607), AKT (Ser473), GSK3β (Ser9), and GS (Ser641) analyzed by Western blot.(*p < 0.05, **p < 0.01, ***p < 0.001, ****p < 0.0001).

RT‐qPCR analysis revealed that mRNA expression levels of *IRS1*, *PI3K*, *AKT*, *GSK3β*, and *GS* were significantly downregulated in the model group compared to the control group (*p* < 0.0001; Figure 3A–E). Sinapine treatment restored their expression in a concentration‐dependent manner.

Specifically, sinapine at all tested concentrations (20–80 μmol/L) significantly upregulated IRS1 (Figure 3A) and PI3K (Figure 3B) mRNA levels (*p* < 0.0001), while AKT (Figure 3C) expression was significantly increased in the medium and high concentration groups (*p* < 0.0001), with no significant change in the low concentration group. GSK3β (Figure 3D) and GS (Figure 3E) mRNA levels were significantly elevated in all sinapine‐treated groups (*p* < 0.05 to *p* < 0.0001), further supporting a concentration‐dependent regulatory effect.

### 3.6 Phosphorylation of Insulin Resistance‐Related Proteins in Sinapine‐Treated HepG2 Cells

Western blot analysis showed that phosphorylation levels of IRS1 (Tyr632), PI3K p85 (Tyr607), AKT (Ser473), and GSK3β (Ser9) were significantly reduced in the model group compared to controls (*p* < 0.0001; Figure 3F–J), indicating impaired insulin signaling.

Sinapine treatment significantly restored phosphorylation of IRS1 (Figure 3F), AKT (Figure 3H), and GSK3β (Figure 3I) at all tested concentrations (*p* < 0.0001), while PI3K‐p85 phosphorylation was significantly improved in the medium and high concentration groups (*p* < 0.0001; Figure 3G), but not in the low concentration group. In contrast, phosphorylation of GS (Ser641; Figure 3J), which was abnormally elevated in the model group, was significantly reduced by sinapine in a concentration‐dependent manner (*p* < 0.0001), suggesting restored insulin sensitivity.

### 3.7 Sinapine Improves Metabolic Parameters Including Body Weight, Blood Glucose, and Liver Index in T2DM Mice

**FIGURE 4:**
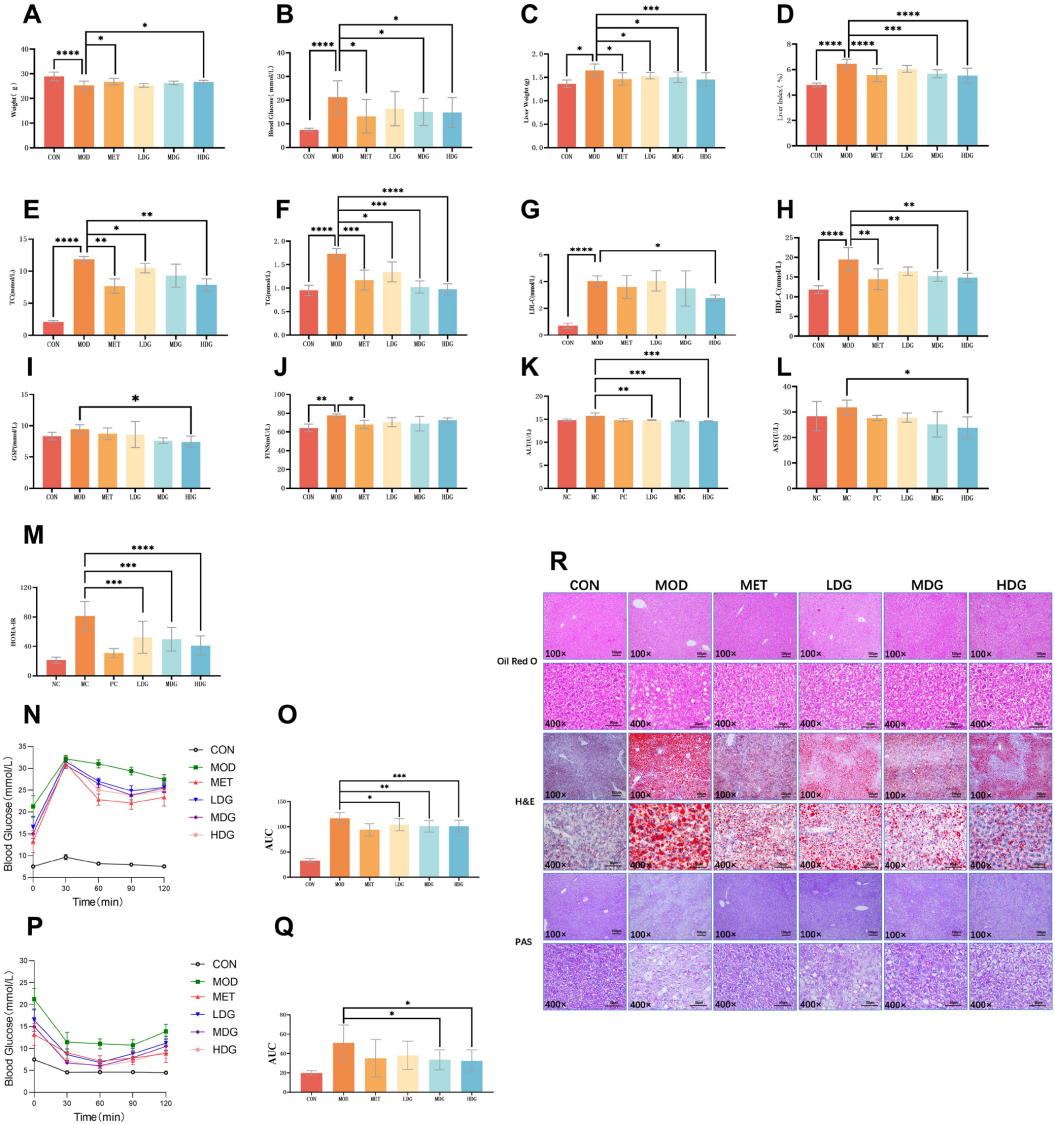
Effects of sinapine on metabolic parameters, glucose and lipid metabolism, and hepatic histopathology in T2DM mice. (A) Body weight; (B) Blood glucose level; (C) Liver weight; (D) Liver index; (E) Serum total cholesterol (TC); (F) Serum triglyceride (TG); (G) Low‐density lipoprotein cholesterol (LDL‐C); (H) High‐density lipoprotein cholesterol (HDL‐C); (I) Glycosylated serum protein (GSP); (J) Fasting insulin (FINS); (K) Alanine aminotransferase (ALT); (L) Aspartate aminotransferase (AST); (M) Homeostasis model assessment of insulin resistance (HOMA‐IR); (N) Oral glucose tolerance test (OGTT) curve; (O) Area under the OGTT curve; (P) Insulin tolerance test (ITT) curve; (Q) Area under the ITT curve; (R) Histological analysis of liver tissue by Oil Red O, HE, and PAS staining. (*p < 0.05, **p < 0.01, ***p < 0.001, **** *p* < 0.0001).

As shown in Figure 4A, body weight in the control group (CON) remained within the normal range. The model group (MOD) exhibited significant weight loss compared to CON (*p* < 0.0001), while metformin‐treated mice (MET) showed a partial recovery of body weight (*p* < 0.05). Among the sinapine‐treated groups, only the high‐dose group (HDG) demonstrated a significant increase in body weight compared to MOD (*p* < 0.05).

After 6 weeks of a high‐fat and high‐sugar diet, fasting blood glucose levels were markedly elevated in the MOD group compared to the CON group (*p* < 0.0001). Both metformin and sinapine (MDG, HDG) significantly reduced fasting blood glucose levels compared to MOD (*p* < 0.05; Figure 4B).

As shown in Figure 4C,D, the MOD group showed significantly increased liver weight and liver index compared to CON (*p* < 0.0001 for both), indicating liver hypertrophy under diabetic conditions. Liver index was significantly lowered in the MDG and HDG groups (*p* < 0.05 and *p* < 0.0001).

### 3.8 Sinapine Ameliorates Lipid Metabolism Disorders in T2DM Mice

As shown in Figure 4E–H, the MOD group exhibited significant elevations in serum TC, TG, LDL‐C, and HDL‐C levels compared to the CON group (*p* < 0.001), indicating notable lipid dysregulation. Sinapine treatment led to dose‐dependent improvements in lipid profiles. All sinapine‐treated groups showed significant reductions in TG levels, and both the MDG and HDG also exhibited significant decreases in TC (*p* < 0.05 and *p* < 0.01). Notably, LDL‐C was significantly reduced only in the HDG group, while HDL‐C levels were significantly decreased in the MDG and HDG groups (*p* < 0.05 or *p* < 0.01).

### 3.9 Sinapine Improves Glucose Metabolism in T2DM Mice

As shown in Figure 4N,O, OGTT results revealed a significant rise in blood glucose levels across all groups after glucose administration, peaking at 30 min and gradually declining thereafter. The MOD group exhibited consistently higher glucose levels and a significantly increased AUC compared to the CON group (*p* < 0.0001), indicating impaired glucose tolerance. Treatment with metformin or sinapine (LDG, MDG, HDG) significantly reduced glucose levels and AUCs (*p* < 0.05 and *p* < 0.01), demonstrating improved glucose tolerance.

Similarly, ITT results (Figure 4P,Q) showed that blood glucose levels in all groups reached a minimum at 60 min post‐insulin injection. The MOD group had a slower decline and higher glucose levels throughout, reflecting insulin resistance. In contrast, metformin and sinapine‐treated groups showed greater reductions in glucose and significantly lower AUCs (*p* < 0.05 or *p* < 0.01), indicating enhanced insulin sensitivity.

Regarding glycated serum protein (GSP, Figure 4I), the MOD group showed increased levels compared to CON, though not statistically significant. While metformin and LDG sinapine groups exhibited non‐significant reductions, HDG significantly decreased GSP levels (*p* < 0.05), suggesting improved long‐term glycemic control.

In terms of fasting insulin (FINS, Figure 4J), MOD mice exhibited significantly elevated levels compared to CON (*p* < 0.01). Metformin treatment significantly reduced FINS levels (*p* < 0.05), whereas sinapine treatment (LDG, MDG, HDG) showed no significant difference from the MOD group. In addition, the homeostasis model assessment of insulin resistance (HOMA‐IR) was employed to further evaluate insulin sensitivity. As shown in Figure 4M, HOMA‐IR was markedly increased in the MOD group compared with the CON group (*p* < 0.001), indicating severe insulin resistance. Sinapine administration significantly reduced HOMA‐IR in diabetic mice, with the LDG and MDG showing decreases relative to the MOD group (*p* < 0.001). Notably, the HDG exhibited a comparable improvement to that observed in the metformin‐treated group (*p* < 0.0001). These results demonstrate that sinapine alleviates insulin resistance and enhances insulin sensitivity in T2D mice.

### 3.10 Histological and Biochemical Improvements in Liver Tissues Induced by Sinapine

Serum alanine aminotransferase (ALT) and aspartate aminotransferase (AST) levels were measured to evaluate the hepatic safety of sinapine treatment (Figure 4K,L). Sinapine treatment reduced the elevated ALT and AST levels in a dose‐dependent manner. ALT levels were significantly decreased in all sinapine‐treated groups compared with the model group, with the high‐dose group showing the greatest reduction (*p* < 0.001). AST levels also decreased following sinapine treatment, with a significant reduction observed only in the high‐dose group (*p* < 0.05). These trends were comparable to those observed in the metformin‐treated group. Collectively, these findings suggest that sinapine does not exert hepatotoxic effects and may help restore hepatic function impaired by diabetes.

Histological analyses using Oil Red O, H&E, and PAS staining (Figure 4R) revealed pronounced hepatic alterations in T2DM mice, which were alleviated by sinapine treatment.

Oil Red O staining showed abundant red‐stained lipid droplets and disrupted hepatic architecture in the MOD group, indicating marked hepatic steatosis.

H&E staining revealed hepatocyte swelling, vacuolar degeneration, and inflammatory infiltration in the MOD group, consistent with fatty liver pathology.

PAS staining demonstrated weakened and uneven glycogen staining in the MOD group, reflecting impaired hepatic glycogen synthesis. Treatment with sinapine restored glycogen distribution and staining intensity to varying degrees. Notably, HDG achieved the most prominent improvement, approaching the efficacy observed with metformin.

### 3.11 Sinapine Activates the PI3K/AKT/GSK3β Signaling Pathway in Liver Tissues of T2DM Mice

**FIGURE 5:**
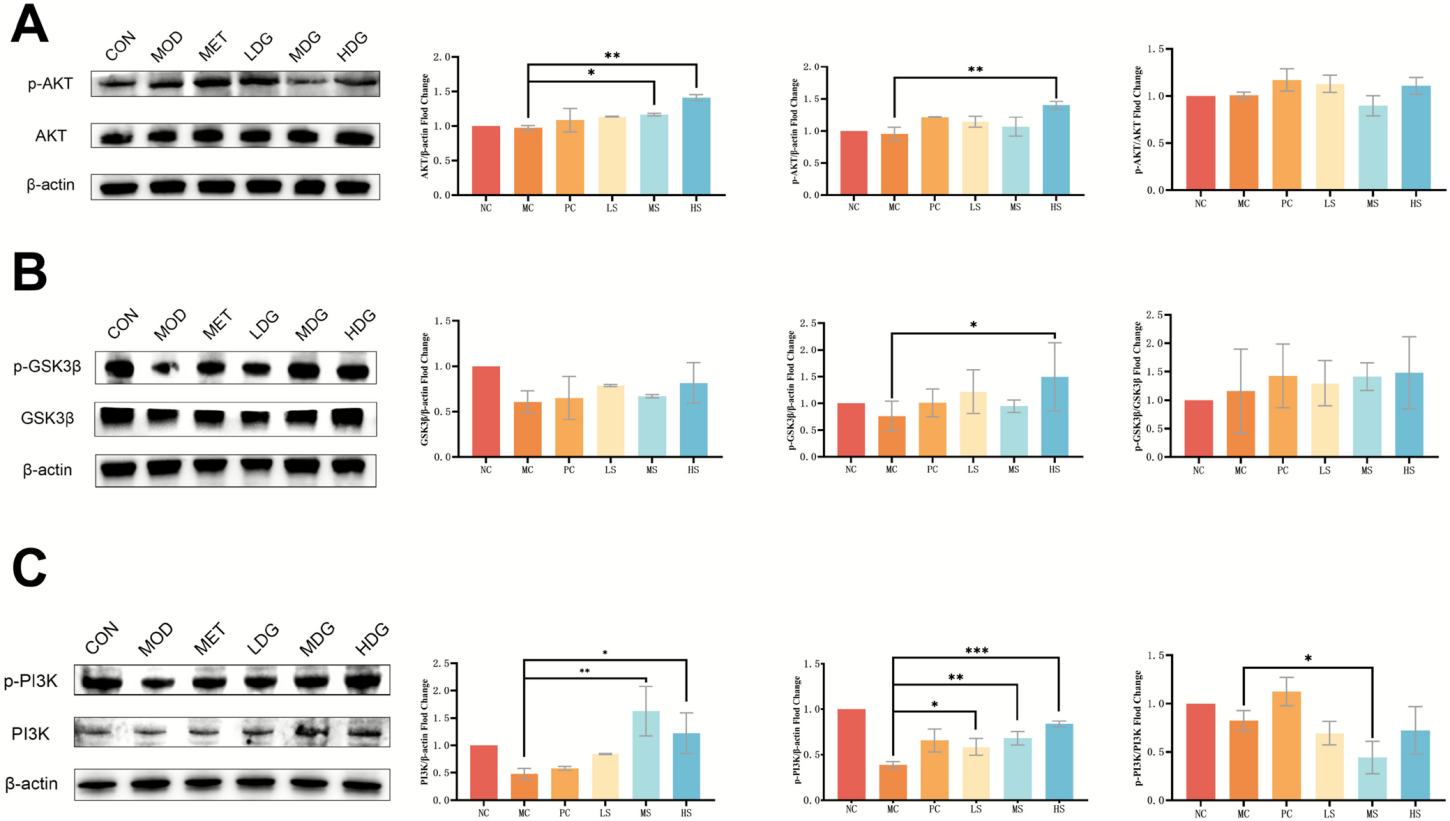
Effects of sinapine treatment on the PI3K/AKT/GSK3‐β signaling pathway in the liver of T2DM mice. (A) Protein expression and relative quantification of phosphorylated and total AKT; (B) Protein expression and relative quantification of phosphorylated and total GSK3‐β; (C) Protein expression and relative quantification of phosphorylated and total PI3K. (*p < 0.05, **p < 0.01, ***p < 0.001, ****p < 0.0001).

Western blot results (Figure 5A–C) suggested modest changes in the PI3K/AKT/GSK3β signaling pathway. For AKT, total protein expression was significantly upregulated in the MDG and HDG compared to the MOD (*p* < 0.05 and *p* < 0.01), indicating enhanced AKT synthesis or stability. Phosphorylated AKT (p‐AKT) levels were significantly increased only in the HDG suggesting that sinapine may promote AKT activation to some extent, albeit not robustly across all doses. In contrast, both total and phosphorylated PI3K levels were significantly elevated in the MDG and HDG groups. Furthermore, the p‐PI3K/PI3K ratio was significantly elevated in the MDG. Results suggest that sinapine may exert its beneficial effects on hepatic insulin signaling primarily through activation of the PI3K arm of the IRS1–PI3K–AKT–GSK3β axis.

## 4 Discussion

This study combined network pharmacology and molecular docking to explore sinapine's targets and mechanisms in improving insulin resistance, followed by validation through in vitro and in vivo experiments. In this study, potential targets and signaling pathways of sinapine were identified using network pharmacology and molecular docking, and then its effects on glucose regulation in IR HepG2 cells and animal models were tested.

Previous studies have shown that modulation of key rate‐limiting enzymes in glycogen synthesis and gluconeogenesis can improve hepatic insulin resistance (Fan et al. 2023). Therefore, in this experiment, an insulin resistance model was performed in HepG2 cells with 10 μmol/L sodium palmitate and 0.1 μmol/L insulin. The effect of sinapine on hepatic glucose metabolism was investigated by evaluating glycogen content, PEPCK and G6Pase enzyme activities, and mRNA and protein expression in IR HepG2 cells. The results showed a significant decrease in glucose uptake and glycogen synthesis in the model cells, accompanied by an increase in the activity of the gluconeogenic enzymes PEPCK and G6Pase. Gluconeogenesis is mainly regulated by PEPCK and G6Pase (Wang et al. 2014). Sinapine effectively inhibits gluconeogenesis and increases insulin sensitivity in the current study.

Experimental results showed a significant improvement in cellular lipid accumulation, as excess adipose tissue releases excessive free fatty acids and ROS, which can accumulate in non‐adipose organs as ectopic fat, ultimately leading to lipotoxicity that exacerbates insulin resistance (Ahmed et al. 2021). ROS, which are formed by the reduction of one or more electrons of oxygen, are considered harmful byproducts of cell metabolism and have significant effects on various signaling pathways (Hernansanz‐Agustín and Enríquez 2021; Sies and Jones 2020). The results of this experiment showed that intervention with sinapine significantly reduced the abnormal increase in intracellular ROS, suggesting that sinapine has potent antioxidant properties and could improve insulin resistance through its antioxidant functions. The results are consistent with previous studies showing that sinapine can effectively enter mitochondria, selectively reduce oxidative stress in mitochondria and significantly limit the production of ROS during cardiac ischemia–reperfusion (Boulghobra et al. 2020).

To further validate the in vitro results, animal experiments were conducted using a T2DM mouse model. Blood glucose and body weight are key indicators that need to be closely monitored in the treatment of diabetes. The intervention with sinapine effectively regulated the impaired glucose metabolism and at the same time mitigated the body weight loss observed in the mice. In this study, the abnormal glucose tolerance of the mice improved after treatment with sinapine, indicating improved glucose tolerance. In addition, the treatment significantly reduced insulin resistance caused by a high‐fat diet and restored insulin sensitivity. Taken together, these results demonstrate that sinapine exerts multi‐level effects on glucose metabolism, including enhanced glucose uptake, improved insulin responsiveness, and preservation of body weight.

Type 2 diabetes is often associated with impaired lipid metabolism, which increases the risk of cardiovascular disease. In this study, mice showed improvements in lipid abnormalities after treatment with sinapine, with the cholesterol ratio being regulated, suggesting that sinapine has lipid‐lowering effects and can modulate lipid metabolism. Serum glycosylated protein (GSP) is one of the diagnostic standards for diabetes and reflects long‐term changes in blood glucose levels. The results show that sinapine inhibits the abnormal GSP levels caused by a high‐fat diet and thereby improves glucose homeostasis in mice. Histologic analysis of liver tissue using various staining techniques showed that sinapine improved the pathological condition of liver tissue. By reversing liver damage, sinapine effectively regulated key indicators such as blood glucose and blood lipids in the T2DM model.

In addition, Western blot analysis showed that sinapine upregulated the phosphorylation levels of key proteins in the IRS1–PI3K–AKT–GSK–GS pathway, promoted glycogen synthesis and inhibited gluconeogenesis. These results were highly consistent with the in vitro data and the whole animal level. Analysis of five target genes in the IRS1–PI3K–AKT–GSK–GS signaling pathway revealed that sinapine upregulated the mRNA expression levels of IRS1, PI3K, AKT, GSK and GS. It increased the phosphorylation of IRS1, PI3K, AKT, and GSK and decreased GS phosphorylation, thereby promoting glycogen synthesis. In normal insulin signaling, IRS phosphorylation leads to binding to the p85 regulatory subunit of PI3K, activating PI3K, which in turn phosphorylates AKT, leading to its full activation (Savova et al. 2023). This process subsequently promotes the phosphorylation of GSK3, inhibits its activity, increases GS expression, and decreases GS phosphorylation, thereby facilitating glycogen synthesis (Żołnierkiewicz and Rogacka 2024). Several previous experiments (Tonks et al. 2013; Vind et al. 2011) have shown that even with decreased phosphorylation of proximal components such as AKT, they can still transmit normal signals without impairing pathway transmission (Meyer et al. 2002). Defects in signaling through distal components such as GSK3 are more likely to be a potential cause of insulin resistance. When studying insulin resistance signaling pathways, it is crucial to closely examine the distal components and their representative functional properties and use the results of the proximal components as supplementary references. GS, which is closely associated with GSK3, regulates hepatic glycogen synthesis, which is essential for glucose metabolism in the liver (Deng et al. 2016).

Phosphorylated AKT can inhibit the expression of the gluconeogenic enzymes G6Pase and PEPCK, thereby reducing gluconeogenesis and jointly regulating blood glucose levels (Kuo et al. 2014). Sinapine attenuates the cytotoxicity of palmitate, promotes the expression of IRS1, PI3K, AKT, GSK3β, and GS proteins, increases the phosphorylation of IRS1, PI3K, AKT and GSK3β proteins, and decreases GS phosphorylation levels, thereby enhancing insulin resistance signaling pathways in hepatocytes, while decreasing the expression of gluconeogenic enzymes G6Pase and PEPCK.

In summary, sinapine was confirmed to improve insulin resistance by upregulating the mRNA expression of IRS1, PI3K, AKT, GSK3β and GS and interacting with the target proteins to affect their phosphorylation. This increases the phosphorylation of IRS1, PI3K, AKT, and GSK3β, while decreasing the phosphorylation of GS, thereby enhancing the transmission of insulin resistance signaling pathways in liver cells. Sinapine also reduces the expression of the gluconeogenic enzymes G6Pase and PEPCK, resulting in less gluconeogenesis.

## Author Contributions

**Tiancheng Xing and Yiling Bai:** data curation, formal analysis, funding acquisition, investigation, writing‐original. **Weijie Wu:** writing, formal analysis, investigation. **Ziqi Zhao:** formal analysis, investigation. **Hanyu Kong:** formal analysis, investigation. **Qianyi Zhang:** formal analysis, investigation. **Shuoqi Li:** formal analysis, investigation. **Xiaohui Guo, Yan Liu, Zengli Wang:** conceptualization, funding acquisition, supervision, writing – review and editing.

## Funding

This work was supported by Beijing Natural Science Foundation (Grant No. 6222023), and Shandong Provincial Key Research and Development Program (Grant No. 2024TSGC0072).

## Conflicts of Interest

The authors declare no conflicts of interest.

## Supporting information


**Table S1:** Materials and instruments.
**Table S2:** Chemicals and reagents.
**Table S3:** Software tools and databases.
**Table S4:** Protein structure and parameters.
**Table S5:** Target genes of sinapine‐insulin resistance.
**Table S6:** Detailed results of Gene Ontology (GO) enrichment analysis.

## Data Availability

The datasets generated and analyzed during the in vitro and in vivo experiments are not publicly available due to intellectual property restrictions by the funding agency, but are available from the corresponding author upon reasonable request. Publicly available datasets were analyzed in this study. The sources and accession numbers of these publicly available datasets are detailed in the Supporting Information of this article.
