# Supplementary material for: Sinapine Modulates Glycogen and Lipid Synthesis via IRS1–PI3K–AKT–GSK3β–GS Pathway in Insulin‐Resistant Models
Source: Food Sci Nutr. 2026 Jan 4;14(1):e71304. doi: 10.1002/fsn3.71304 (PMC12765658; doi:10.1002/fsn3.71304)
Supplement: Supplementary file 1 — Table S1: Materials and instruments. Table S2: Chemicals and reagents. Table S3: Software tools and databases. Table S4: Protein structure and parameters. Table S5: Target genes of sinapine‐insulin resistance. Table S6: Detailed results of Gene Ontology (GO) enrichment analysis. [file FSN3-14-e71304-s001.docx]

***Supplementary materials for***

***Investigation of the effects of sinapine on glycogen and lipid synthesis in vivo and in vitro insulin-resistance models via the IRS1-PI3K-AKT-GSK-GS pathway.***

Tiancheng Xing, Ziqi Zhao, Hanyu Kong, Weijie Wu, Qianyi Zhang, Yiling Bai, Shuoqi Li, Zengli Wang, Xiaohui Guo*

College of Food Science and Nutritional Engineering, China Agricultural University, Beijing 100083, China

*Corresponding author:

Xiaohui Guo, E-mail: [guoxiaohui@cau.edu.cn](mailto:guoxiaohui@cau.edu.cn)

**Table S1** Materials and Instruments

| **Item** | **Supplier / Manufacturer** | **Model / Catalog No.** | **Notes** |
| --- | --- | --- | --- |
| HepG2 human liver cancer cells | Institute of Biophysics, Chinese Academy of Sciences | – | Cell line |
| Sinapine thiocyanate (≥98%, HPLC) | Solarbio Biotechnology Co., Ltd. | – | Compound |
| Cell culture plates (96/24/6-well) | Corning, USA | – | Cell culture |
| Culture flasks | Corning, USA | – | Cell culture |
| Centrifuge tubes (15 mL, 50 mL) | Corning, USA | – | General labware |
| Cryovials (2 mL) | Corning, USA | – | Sample storage |
| Sealing foils | Solarbio Biotechnology Co., Ltd. | PM996 | Plate sealing |
| Microplate reader | Thermo Fisher Scientific | Multiskan FC | Absorbance measurements |
| Medical centrifuge | Hunan Hengnuo Instrument Equipment Co., Ltd. | TD6 | Cell/solution centrifugation |
| Inverted fluorescence microscope | Olympus (China) Co., Ltd. | BX63 | Microscopy |
| Fluorescence microplate reader | BMG LABTECH | FLUOstar Omega | Fluorescence measurements |
| Saline solution (0.9%, sterile) | Solarbio Biotechnology Co., Ltd. | IN9000 | Animal experiments |
| PVDF membrane | Millipore, USA | – | Protein transfer |

**Table S2** Chemicals and Reagents

| **Reagent / Chemical / Antibody** | **Supplier** | **Catalog No. (if available)** | **Notes** |
| --- | --- | --- | --- |
| DMEM (high glucose) | Gibco, USA | – | Cell culture medium |
| Penicillin-streptomycin mixed solution | Gibco, USA | – | Cell culture supplement |
| Fetal bovine serum (FBS) | Corning, USA | 35-081-CV | For cell culture |
| High quality FBS (phage-free, low endotoxin) | Sijiqing Company | – | Alternative FBS |
| Dimethyl sulfoxide (DMSO, cell grade) | Solarbio Biotechnology Co., Ltd. | – | Solvent |
| Trypsin-EDTA (0.25%) | Solarbio Biotechnology Co., Ltd. | T300 | Cell digestion |
| Glycogen content detection kit | Solarbio Biotechnology Co., Ltd. | BC0345 | Biochemical assay |
| Glycogen content detection kit | Shanghai Yuanxin Biotechnology Co., Ltd. | YX-E-N326 | For animal samples |
| Oil Red O (saturated solution, analytical grade) | Solarbio Biotechnology Co., Ltd. | – | Lipid staining |
| PEPCK activity detection kit | Solarbio Biotechnology Co., Ltd. | BC3310 | Enzyme activity assay |
| G6P activity detection kit | Solarbio Biotechnology Co., Ltd. | BC3320 | Enzyme activity assay |
| Phosphate-buffered saline (PBS, cell grade) | Beyotime Biotechnology | – | Cell washing solution |
| Glucose detection kit (O-toluidine method) | Beyotime Biotechnology | – | Biochemical assay |
| Cell proliferation and toxicity detection kit (CCK-8) | Yuan Ye Biotechnology Co., Ltd. | R22305-500T | Cell viability assay |
| Isopropanol (analytical grade) | Jingchun Reagent Co., Ltd. | – | Staining/washing |
| Reactive Oxygen Species (ROS) detection kit | Wuhan Sevier Biotechnology Co., Ltd. | G1706 | Fluorescent probe |
| Citric acid (≥99.5%) | Solarbio Biotechnology Co., Ltd. | – | Animal experiments |
| Sodium citrate (≥98%) | Solarbio Biotechnology Co., Ltd. | – | Animal experiments |
| Streptozotocin (STZ) | Solarbio Biotechnology Co., Ltd. | S8051-100mg | Inducing diabetes |
| Metformin hydrochloride (≥98%, HPLC) | Solarbio Biotechnology Co., Ltd. | – | Positive control drug |
| 20% Glucose solution | Solarbio Biotechnology Co., Ltd. | – | Supplement after STZ injection |
| Insulin (NovoRapid, 100 U/mL) | Novo Nordisk (China) Pharmaceuticals | – | Animal treatment |
| 10% Formalin solution (RNase-free) | Solarbio Biotechnology Co., Ltd. | IF9010 | Tissue fixation |
| PAS Staining Kit (with Hematoxylin) | Solarbio Biotechnology Co., Ltd. | G1281 | Glycogen staining |
| Hematoxylin and Eosin (HE) Staining Kit | Solarbio Biotechnology Co., Ltd. | G1120 | Histology |
| Triglyceride (TG) Assay Kit | Nanjing Jiancheng Institute | BC0625 | Biochemical assay |
| Total Cholesterol (T-CHO) Assay Kit | Nanjing Jiancheng Institute | BC1985 | Biochemical assay |
| LDL-C Assay Kit | Nanjing Jiancheng Institute | BC5335 | Biochemical assay |
| HDL-C Assay Kit | Nanjing Jiancheng Institute | BC5325 | Biochemical assay |
| Glycated Serum Protein (GSP) Assay Kit | Nanjing Jiancheng Institute | BC4945 | Biochemical assay |
| RIPA Lysis Buffer | Solarbio Biotechnology Co., Ltd. | – | Protein extraction |
| Protease and phosphatase inhibitor cocktail | Solarbio Biotechnology Co., Ltd. | – | For protein protection |
| Primary antibody diluent | Solarbio Biotechnology Co., Ltd. | – | For western blotting |
| TBST buffer | Solarbio Biotechnology Co., Ltd. | – | Western blot buffer |
| BCA Protein Assay Kit (enhanced) | Solarbio Biotechnology Co., Ltd. | – | Protein quantification |
| ECL Chemiluminescence Detection Kit | Solarbio Biotechnology Co., Ltd. | – | Western blot detection |
| Anti-p-PI3K (p85α)-Tyr607 antibody | Abmart Pharmaceuticals (Shanghai) | – | Primary antibody |
| Anti-PI3Kα antibody | Jiangsu Qingke Biological Center | – | Primary antibody |
| Anti-Akt antibody | Wuhan Sanying Biotechnology | – | Primary antibody |
| Anti-p-Akt-Ser473 antibody | Wuhan Sanying Biotechnology | – | Primary antibody |
| Anti-GSK3β antibody | Wuhan Sanying Biotechnology | – | Primary antibody |
| Anti-GSK3β-Ser9 antibody | Wuhan Sanying Biotechnology | – | Primary antibody |
| β-actin antibody | Wuhan Sanying Biotechnology | – | Loading control antibody |
| HRP-conjugated goat anti-mouse secondary antibody | Wuhan Sanying Biotechnology | – | Secondary antibody |
| HRP-conjugated goat anti-rabbit secondary antibody | Wuhan Sanying Biotechnology | – | Secondary antibody |

**Table S3** Software Tools and Databases

| **Name** | **Website URL** | **Function / Application** |
| --- | --- | --- |
| PubChem | <https://pubchem.ncbi.nlm.nih.gov/> | Chemical information |
| SwissADME | <http://www.swissadme.ch/> | ADME prediction |
| BATMAN-TCM | <http://bionet.ncpsb.org.cn/batman-tcm/> | TCM target prediction |
| PharmMapper | <http://www.lilab-ecust.cn/pharmmapper/> | Pharmacophore mapping |
| TCMSP | <https://tcmsp-e.com/> | TCM compound database |
| HERB | <http://herb.ac.cn/> | Herbal molecular mechanism analysis |
| OMIM | <https://omim.org/> | Genetic disease database |
| STRING | <https://www.string-db.org/> | Protein-protein interaction network |
| RCSB PDB | <https://www.rcsb.org/> | Protein structure database |
| UniProt | <https://www.uniprot.org/> | Protein sequence database |
| CB-DOCK2 | <https://cadd.labshare.cn/cb-dock2/php/index.php> | Molecular docking |
| AutoDock Vina | <https://autodock.scripps.edu/> | Molecular docking software |
| LigPlot+ | <https://www.ebi.ac.uk/thornton-srv/software/LigPlus/> | Protein-ligand interaction visualization |
| PyMOL | <https://pymol.org/2/> | 3D structure visualization |
| Cytoscape | <https://cytoscape.org/> | Network visualization |
| R | <https://cloud.r-project.org/> | Statistical computing |

| **Target Name** | **UniProtKB** | **PDB ID** | **Resolution** | **Molecular Weight（kDA）** | **Atomic Number** |
| --- | --- | --- | --- | --- | --- |
| AKT1 | P31749 | 1H10 | 1.40Å | 15.44 | 1266 |
| AKT2 | P31751 | 1GZK | 2.30Å | 36.49 | 2481 |
| AKT3 | Q9Y243 | 2X18 | 1.46Å | 115.26 | 9414 |
| GSK3A | P49840 | 7SXF | 1.94Å | 41.66 | 2931 |
| GSK3B | P49841 | 1H8F | 2.80Å | 80.08 | 5786 |
| GS | P13807 | 7Q0B | 3.00Å | 493.39 | 21168 |
| INSR | P06213 | 1GAG | 2.70Å | 36.99 | 2520 |
| IRS1 | P35568 | 1IRS | 2.30 Å | 13.89 | 971 |
| PI3K | P42336 | 7PG5 | 2.20 Å | 159.1 | 10787 |
| PIK3CA | P42336 | 2RD0 | 3.05Å | 161.49 | 9365 |
| PIK3CD | O00329 | 5DXU | 2.64 Å | 140.76 | 8955 |

**Table S4** Protein structure and parameters

**Table S5** Target genes of sinapine-insulin resistance

| Target Genes Name | | | | | | | | | |  |
| --- | --- | --- | --- | --- | --- | --- | --- | --- | --- | --- |
| ABL1 | ACACB | ACE | ACHE | | ADORA1 | | ADRB1 | | | |
| ADRB2 | AGPAT2 | AKT1 | ALDH2 | | AURKA | | BCHE | | | |
| CASP1 | CASP3 | CCL5 | CDK4 | | CFTR | | CNR1 | | | |
| CTSB | CTSD | DRD1 | DRD2 | | EGFR | | ENPP1 | | | |
| ESR1 | ESR2 | FASN | FGFR1 | | FLT3 | | GCGR | | | |
| GSK3A | GSK3B | GSTM1 | GSTP1 | | HIF1A | | IGF1R | | | |
| INSR | JAK2 | KCNMA1 | KCNMB1 | | KIT | | MAPK8 | | | |
| MET | MIF | MMP9 | MTOR | | NOS2 | | PARP1 | | | |
| PDE4D | PDGFRB | PDPK1 | PIK3CA | | PIK3CD | | PPARA | | | |
| PRKCA | PRKCB | PRKCD | PRKCE | | PRKCQ | | PRKCZ | | | |
| PTK2 | RAC1 | RAF1 | RPS6KA3 | | | RXRA | | SCD |  |  |
| SLC5A2 | SRC | SREBF2 | | STAT1 | | THRB | | TYMS |  |  |

| **GO Category** | **ID** | **GeneRatio** | **BgRatio** | **pvalue** | **p.adjust** | **qvalue** | **Count** |
| --- | --- | --- | --- | --- | --- | --- | --- |
| BP | GO:0018209 | 22/72 | 333/18866 | 1.08E-21 | 3.71E-18 | 1.57E-18 | 22 |
|  | GO:0018105 | 21/72 | 310/18866 | 6.23E-21 | 1.07E-17 | 4.52E-18 | 21 |
|  | GO:0071375 | 20/72 | 330/18866 | 5.54E-19 | 4.11E-16 | 1.74E-16 | 20 |
|  | GO:0043434 | 22/72 | 447/18866 | 6.29E-19 | 4.11E-16 | 1.74E-16 | 22 |
|  | GO:0032868 | 19/72 | 283/18866 | 6.98E-19 | 4.11E-16 | 1.74E-16 | 19 |
|  | GO:0046777 | 18/72 | 237/18866 | 7.19E-19 | 4.11E-16 | 1.74E-16 | 18 |
|  | GO:1901653 | 21/72 | 398/18866 | 1.1E-18 | 5.39E-16 | 2.28E-16 | 21 |
|  | GO:0018108 | 20/72 | 374/18866 | 6.46E-18 | 2.77E-15 | 1.17E-15 | 20 |
|  | GO:0018212 | 20/72 | 377/18866 | 7.55E-18 | 2.88E-15 | 1.22E-15 | 20 |
|  | GO:0032869 | 17/72 | 226/18866 | 8.89E-18 | 3.05E-15 | 1.29E-15 | 17 |
| CC | GO:0045121 | 9/72 | 329/19559 | 3.24E-06 | 0.000315 | 0.000197 | 9 |
|  | GO:0098857 | 9/72 | 330/19559 | 3.32E-06 | 0.000315 | 0.000197 | 9 |
|  | GO:0098589 | 9/72 | 343/19559 | 4.55E-06 | 0.000315 | 0.000197 | 9 |
|  | GO:0016324 | 9/72 | 361/19559 | 6.88E-06 | 0.000315 | 0.000197 | 9 |
|  | GO:0098978 | 9/72 | 361/19559 | 6.88E-06 | 0.000315 | 0.000197 | 9 |
|  | GO:0031252 | 9/72 | 421/19559 | 2.34E-05 | 0.000892 | 0.000557 | 9 |
|  | GO:0045177 | 9/72 | 433/19559 | 2.91E-05 | 0.000953 | 0.000596 | 9 |
|  | GO:0101002 | 5/72 | 124/19559 | 9.41E-05 | 0.002394 | 0.001497 | 5 |
|  | GO:1904813 | 5/72 | 124/19559 | 9.41E-05 | 0.002394 | 0.001497 | 5 |
|  | GO:0099056 | 4/72 | 74/19559 | 0.00016 | 0.003661 | 0.002289 | 4 |
| MF | GO:0004713 | 13/72 | 135/18352 | 5.02E-15 | 1.97E-12 | 1.27E-12 | 13 |
|  | GO:0004674 | 18/72 | 435/18352 | 5.03E-14 | 9.89E-12 | 6.39E-12 | 18 |
|  | GO:0043560 | 6/72 | 10/18352 | 6.11E-13 | 8.01E-11 | 5.17E-11 | 6 |
|  | GO:0004697 | 6/72 | 16/18352 | 2.29E-11 | 1.8E-09 | 1.16E-09 | 6 |
|  | GO:0004698 | 6/72 | 16/18352 | 2.29E-11 | 1.8E-09 | 1.16E-09 | 6 |
|  | GO:0004714 | 8/72 | 61/18352 | 9.38E-11 | 6.15E-09 | 3.97E-09 | 8 |
|  | GO:0009931 | 6/72 | 23/18352 | 2.82E-10 | 1.58E-08 | 1.02E-08 | 6 |
|  | GO:0010857 | 6/72 | 24/18352 | 3.75E-10 | 1.84E-08 | 1.19E-08 | 6 |
|  | GO:0019199 | 8/72 | 80/18352 | 8.71E-10 | 3.8E-08 | 2.45E-08 | 8 |
|  | GO:0008144 | 8/72 | 104/18352 | 7.18E-09 | 2.82E-07 | 1.82E-07 | 8 |

**Table S6** Detailed results of Gene Ontology (GO) enrichment analysis
